# Milk ladder versus early oral immunotherapy in infants with cow's milk protein allergy

**DOI:** 10.1002/clt2.12388

**Published:** 2024-08-08

**Authors:** Yurika Matsumoto, Mayumi Fujita, Tsukahara Ayumi, Tetsuya Takamasu, Chisato Inuo

**Affiliations:** ^1^ Department of Allergy Kanagawa Children's Medical Center Kanagawa Japan

To the Editor,

Cow's milk protein allergy (CMPA) significantly decreases the quality of life of infants and their families, necessitating effective management. Avoidance of cow's milk protein (CMP) has been the primary approach, awaiting the development of a natural tolerance.^1^

Oral immunotherapy (OIT) for CMPA gradually increases the use of pure CMP, such as milk, to enhance tolerance. A previous study demonstrated the efficacy and safety of early OIT (E‐OIT) for infants with CMPA.^2^ The Milk Ladder (ML) method modifies the OIT strategy and enhances CMP tolerance through stepwise exposure to milk‐containing foods.^1^, ^3^, ^4^, ^5^ Despite growing adoption, ML lacks extensive validation and requires further research.^4^ To the best of our knowledge, there have been no comparative studies of E‐OIT and ML in infants with CMPA. This study aimed to compare the efficacy and safety of ML and E‐OIT in infants with CMPA.

We retrospectively analyzed infants younger than 2 years who started intervention for CMPA at the Department of Allergy at Kanagawa Children's Medical Center from April 2016 to March 2022, with a treatment protocol shift in April 2018 from E‐OIT to ML. Inclusion criteria included a CMPA diagnosis based on parent‐reported immediate allergic reaction to CMP ingestion or a serum milk‐specific IgE level greater than 5 kU_A_/L.^3^ Patients with only gastrointestinal symptoms were excluded due to different CMPA types. The ML protocol started with baked milk (BM), advancing toward less processed forms, while E‐OIT began with controlled doses of milk or yogurt. For detailed protocols, see the Supplementary materials in Supporting Information S1.

CMP tolerance was defined as the ability to consume 100 mL of milk or an equivalent amount of approximately 3300 mg of CMP daily without experiencing symptoms. Low dairy tolerance for processed foods was defined as the ability to consume processed foods. Products high in dairy ingredients, such as cheese, yogurt, and pizza were excluded. These were confirmed through repeated intake at home. All patients underwent treatment review and assessment through interviews approximately every 3 months to assess progress and adjust care, as necessary.

This study conformed to the guidelines established by the Declaration of Helsinki and was approved by the Kanagawa Children's Medical Center Research Ethics Committee (approval no. 2105‐4). Informed consent was obtained from the parents of all patients.

The analyses were performed according to the intention‐to‐treat principle (ITT) 2 years post intervention. The Mann–Whitney *U* test was used to compare continuous variables, while the Chi‐squared test or Fisher's exact test was used for categorical variables. To evaluate the progression of CMP tolerance over time, Kaplan–Meier analysis with a log‐rank test was performed. Statistical significance was set at *p* < 0.05. Statistical analyses were performed using GraphPad Prism 10.2.2 (GraphPad Software Inc.).

We analyzed 89 patients (ML, 38; E‐OIT, 51). The ML group had higher baseline CM‐specific IgE levels and was younger. Patients with a history of anaphylaxis were exclusively found in the ML group (Table 1).

**TABLE 1:** Details of patients and outcomes of the baseline and follow‐up studies.

	Milk ladder	Early oral immunotherapy	*p* value
Background			
Number of patients	38	51	
Male (%)	26 (68)	32 (63)	0.65
Median age, years (IQR)	1.208 (0.67–2)	1.417 (0.5–1.91)	0.008
Other food allergies (%)	21 (55)	26 (51)	0.52
Atopic dermatitis (%)	29 (76)	32 (63)	0.16
Bronchial asthma (%)	2 (5)	3 (6)	>0.99
History of milk anaphylaxis (%)	3 (8)	0	0.07
Cow's milk sIgE (kU_A_/L), median (IQR)	19.35 (0.91–89.5)	8.04 (0.13–73.3)	0.008
Casein sIgE (kU_A_/L), median (IQR)	17.90 (0.22–100)	6.89 (0.08–100)	0.07
Outcome			
Cow's milk protein tolerance (%)	18 (47)	18 (35)	0.28
Processed foods with low amount of dairy tolerance (%)	29 (76)	25 (49)	0.007
Protocol discontinuation	6 (16)	14 (27)	0.21
Lost to follow‐up (%)	4 (11)	6 (12)	>0.99
Refused continuation of treatment (%)	2 (5)	8 (16)	0.18
Changed treatment regimen (%)	5 (13)	4 (8)	0.49
Adverse events			
Total number of symptoms (%)	21 (55)	32 (63)	0.51
Skin symptoms (%)	18 (47)	24 (47)	>0.99
Respiratory symptoms (%)	1 (3)	7 (14)	0.13
Gastrointestinal symptoms (%)	1 (3)	3 (6)	0.63
Anaphylaxis (%)	1 (3)	0	0.42
Total number of treatments required (%)	6 (16)	9 (18)	>0.99
Antihistamines (%)	5 (13)	9 (18)	0.34
Steroids (%)	1 (3)	0	0.42
*β*_2_ agonist inhalation (%)	1 (3)	2 (4)	>0.99
Intramuscular adrenaline (%)	1 (3)	0	0.42
Accidental ingestion			
Total number of symptoms (%)	6 (16)	12 (24)	0.43
Skin symptoms (%)	5 (13)	10 (20)	0.56
Respiratory symptoms (%)	1 (3)	4 (8)	0.38
Gastrointestinal symptoms (%)	0	0	0
Anaphylaxis (%)	1 (3)	0	0.42

Abbreviation: sIgE, specific IgE.

The ML and E‐OIT groups showed no significant difference in CMP tolerance (*p* = 0.28). However, the ML group exhibited higher tolerance to processed foods with low amounts of dairy products than the E‐OIT group (*p* = 0.007). On Kaplan–Meier analysis, there was no difference in CMP tolerance over time (Figure 1A; *p* = 0.29), although a significantly lower avoidance of CMP‐containing products was observed in the ML group (Figure 1B; *p* = 0.0030).

**FIGURE 1:**
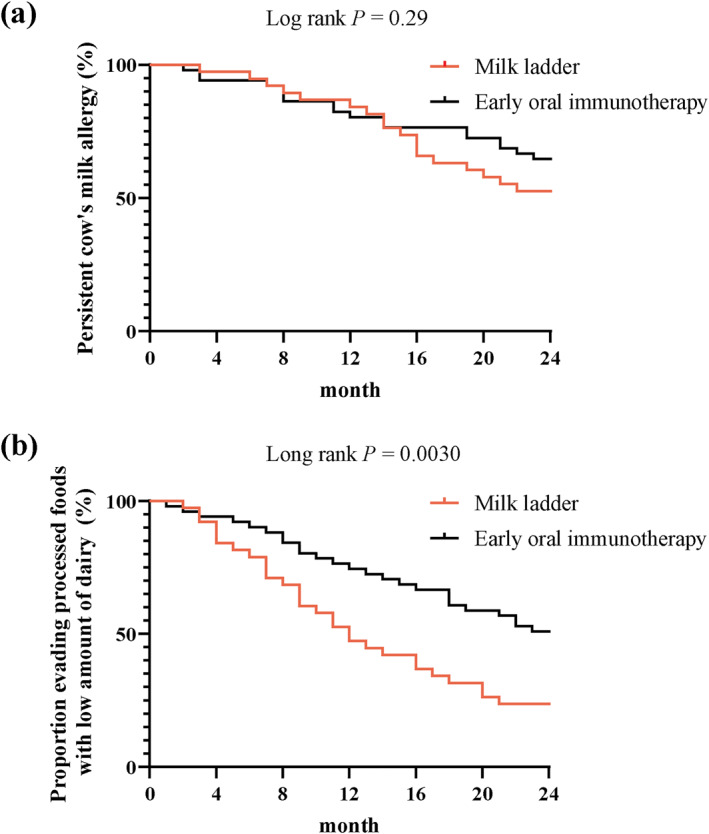
Kaplan–Meier analysis results of (A) the rate of milk allergy persistence in the E‐OIT and ML groups, and (B) the rate of avoidance of processed foods with low amounts of dairy in the E‐OIT and ML groups. No change in CMP tolerance was observed over time (Figure 1A; *p* = 0.29); however, the rate of avoidance of CMP‐containing products was much lower in the ML than in the E‐OIT group (Figure 1B; *p* = 0.0030).

Adverse event rates were similar in both groups (*p* = 0.51). One case of anaphylaxis was reported in the ML group. The affected child initially started the ML protocol with BM but discontinued it owing to taste issues. Consequently, the child deviated from the ML group and was switched to yogurt intake, which induced anaphylaxis.

This result suggests that the contribution of ML to CMP tolerance is the same as that of E‐OIT, which is consistent with a previous report indicating that low‐dose OIT may provide a similar therapeutic effect with much greater safety than conventional OIT.^6^ Furthermore, ML facilitates the intake of processed foods, such as bread, cookies, ham, chocolate, and butter, better than E‐OIT. An elimination diet affects the meals of the entire family, necessitating all members to follow dietary restrictions and experience dietary monotony.^7^ The ability to consume processed products containing milk protein without allergic symptoms may alleviate anxiety about hidden allergens and reduce the family burden.^4^ Our safety data are consistent with those of previous ML reports.^4^, ^5^, ^8^ The use of food ladders is often associated with isolated skin reactions. However, the sole anaphylaxis case in the ML group underscores the need for careful monitoring of dietary interventions. Considering the high rate of protocol discontinuation among both groups, further improvements in the methods are required for the food ladder.^9^

Our study has several limitations. First, the sample size was small, lacking randomized and blinding. We analyzed the data using the ITT technique to reduce selection bias. The ML group had higher baseline CM‐specific IgE levels and was younger, potentially influencing the result. Prospective, randomized, blinded studies are warranted. Second, we did not conduct a double‐blind oral food challenge test, which is the gold standard for diagnosing and tolerance CMPA. Third, this was a single‐center study, and the results cannot be generalized to other populations.

In conclusion, ML is as effective and safe as E‐OIT for the management of CMPA in infants. Furthermore, ML facilitates the intake of processed foods in infants with CMPA better than E‐OIT.

## AUTHOR CONTRIBUTIONS

**Yurika Matsumoto**: Writing – original draft; investigation; formal analysis. **Mayumi Fujita**: Investigation; data curation; supervision. **Tsukahara Ayumi**: Investigation; data curation. **Tetsuya Takamasu**: Investigation. **Chisato Inuo**: Conceptualization; writing – review & editing; funding acquisition; investigation; supervision; project administration; visualization.

## CONFLICT OF INTEREST STATEMENT

The authors have no conflict of interest to declare.

## Supporting information

Supporting Information S1

## Data Availability

All data generated or analyzed during this study are included in this article. Further inquiries can be directed to the corresponding authors.

## References

[1] Meyer R , Venter C , Bognanni A , et al. World allergy organization (WAO) diagnosis and rationale for action against cow’s milk allergy (DRACMA) guideline update ‐ VII – milk elimination and reintroduction in the diagnostic process of cow’s milk allergy. World Allergy Organ J. 2023;16(7):100785. 10.1016/j.waojou.2023.100785 37546235 PMC10401347

[2] Berti I , Badina L , Cozzi G , et al. Early oral immunotherapy in infants with cow’s milk protein allergy. Pediatr Allergy Immunol. 2019;30(5):572‐574. 10.1111/pai.13057 30950113

[3] Luyt D , Ball H , Makwana N , et al. BSACI guideline for the diagnosis and management of cow’s milk allergy. Clin Exp Allergy. 2014;44(5):642‐672. 10.1111/cea.12302 24588904

[4] Chomyn A , Chan ES , Yeung J , et al. Canadian food ladders for dietary advancement in children with IgE‐mediated allergy to milk and/or egg. Allergy Asthma Clin Immunol. 2021;17(1):83. 10.1186/s13223-021-00583-w 34353372 PMC8340453

[5] Ah HT , Cronin C , Flores L , et al. Safety and effectiveness of a milk ladder for managing children with IgE‐mediated milk allergy. Clin Exp Allergy. 2024;54(1):61‐63. 10.1111/cea.14419 37944550

[6] Miyaji Y , Yamamoto‐Hanada K , Yang L , Fukuie T , Narita M , Ohya Y . Effectiveness and safety of low‐dose oral immunotherapy protocols in paediatric milk and egg allergy. Clin Exp Allergy. 2023;53(12):1307‐1309. 10.1111/cea.14400 37771064

[7] Polloni L , Toniolo A , Lazzarotto F , et al. Nutritional behavior and attitudes in food allergic children and their mothers. Clin Transl Allergy. 2013;3(1):41. 10.1186/2045-7022-3-41 24325875 PMC3878898

[8] Athanasopoulou P , Deligianni E , Dean T , Dewey A , Venter C . Use of baked milk challenges and milk ladders in clinical practice: a worldwide survey of healthcare professionals. Clin Exp Allergy. 2017;47(3):430‐434. 10.1111/cea.12890 28109173

[9] Meyer R , Nowak‐Wegrzyn A . Food allergy ladders: when to use them? Ann Allergy Asthma Immunol. 2024;132(3):263‐264. 10.1016/j.anai.2023.11.026 38056525

