# Supplementary material for: Milk ladder versus early oral immunotherapy in infants with cow's milk protein allergy
Source: Clin Transl Allergy. 2024 Aug 8;14(8):e12388. doi: 10.1002/clt2.12388 (PMC11309850; doi:10.1002/clt2.12388)
Supplement: Supplementary file 1 — Supporting Information S1 [file CLT2-14-e12388-s001.docx]

**Supplementary materials**

**MATERIALS AND METHODS**

**1. Protocol for Milk Ladder (ML)**

The instruction sheet for patients following our milk ladder protocol is shown in Figure S1. Our protocol consists of five steps, which are designed based on the quantity and allergenicity of cow's milk protein. Initially, the patient begins by consuming a minuscule amount of baked milk (BM) containing protein equivalent to 0.06 mL of milk. Subsequently, the BM dose is gradually increased at home. After demonstrating the ability to tolerate a certain amount of BM without any allergic symptoms, the patient is asked to advance to the next step under the guidance of their attending physician. At each successive step, the patient is introduced to a variety of milk-containing foods with increasing allergen content and allergenicity.

**2. Protocol for early oral immunotherapy (E-OIT)**

The standard protocol is presented in Table S1. The patient starts to intake a tiny amount of cow’s milk. Thereafter, the cow milk dose is gradually increased at home. The amount of milk is then gradually increased at the next step if the patient exhibits no allergic symptoms for one week. The starting dose and increment intervals of this protocol were occasionally adjusted for each patient by the attending physician.

3. Timelines of intervention and follow-up in milk ladder and early oral immunotherapy

The intervention for CMPA was initiated for each patient individually at the Department of Allergy at Kanagawa Children's Medical Center between April 2016 and March 2022. In April 2018, our department transitioned the treatment protocol from E-OIT to ML. Patients in both OIT and ML were followed for two years post-intervention. At the 24-month endpoint, we analyzed the participants' tolerance, and those who developed tolerance to milk were no longer followed. Their progress is summarized in Figure S2.

**4. Outcome Assessment**

CMP tolerance was defined as the ability to consume 100 mL of milk or an equivalent amount of approximately 3300 mg of CMP daily without experiencing symptoms. Low dairy tolerance for processed foods was defined as the ability to consume processed foods. Products high in dairy ingredients, such as cheese, yogurt, and pizza were excluded. Patients who could consume the processed dairy products listed in Steps 1, 2, and 3 of our milk ladder (Supplementary Figure 1) without any allergic symptoms were considered to be tolerant of processed foods with low amount of dairy. That tolerance was confirmed through their repeated intake at home. In addition, we compared the adverse events and discontinuations of intervention during that period.

**5. Statistical analysis**

Results for non-normally distributed continuous variables were expressed as medians and interquartile ranges. Statistical analyses were carried out with the GraphPad Prism 10.23 (GraphPad Software Inc., San Diego, CA, USA), and p-values of <0.05 were considered significant. Mann–Whitney U tests were used for intergroup analysis of the same parameters of the background among both groups.

Fisher exact tests were used for comparisons among both groups, such as tolerance rate, adverse events, and discontinuations of intervention. The rate of acquisition of persistent milk allergy and proportion avoiding milk-containing products was analyzed using Kaplan–Meier survival curves with log-rank test for differences between curves. We used intention-to-treat analysis and counted cases that discontinued treatment, switched treatment, or were lost to follow-up as non-tolerance cases.

**Supplementary Table 1**. Standard protocol for early Oral immunotherapy (E-OIT).

| Step | Milk(mL) |
| --- | --- |
| 1 | 0.02 |
| 2 | 0.06 |
| 3 | 0.1 |
| 4 | 0.15 |
| 5 | 0.2 |
| 6 | 0.3 |
| 7 | 0.5 |
| 8 | 0.7 |
| 9 | 1 |
| 10 | 1.5 |
| 11 | 2 |
| 12 | 3 |
| 13 | 5 |
| 14 | 8 |
| 15 | 12 |
| 16 | 15 |
| 17 | 20 |
| 18 | 25 |
| 19 | 30 |


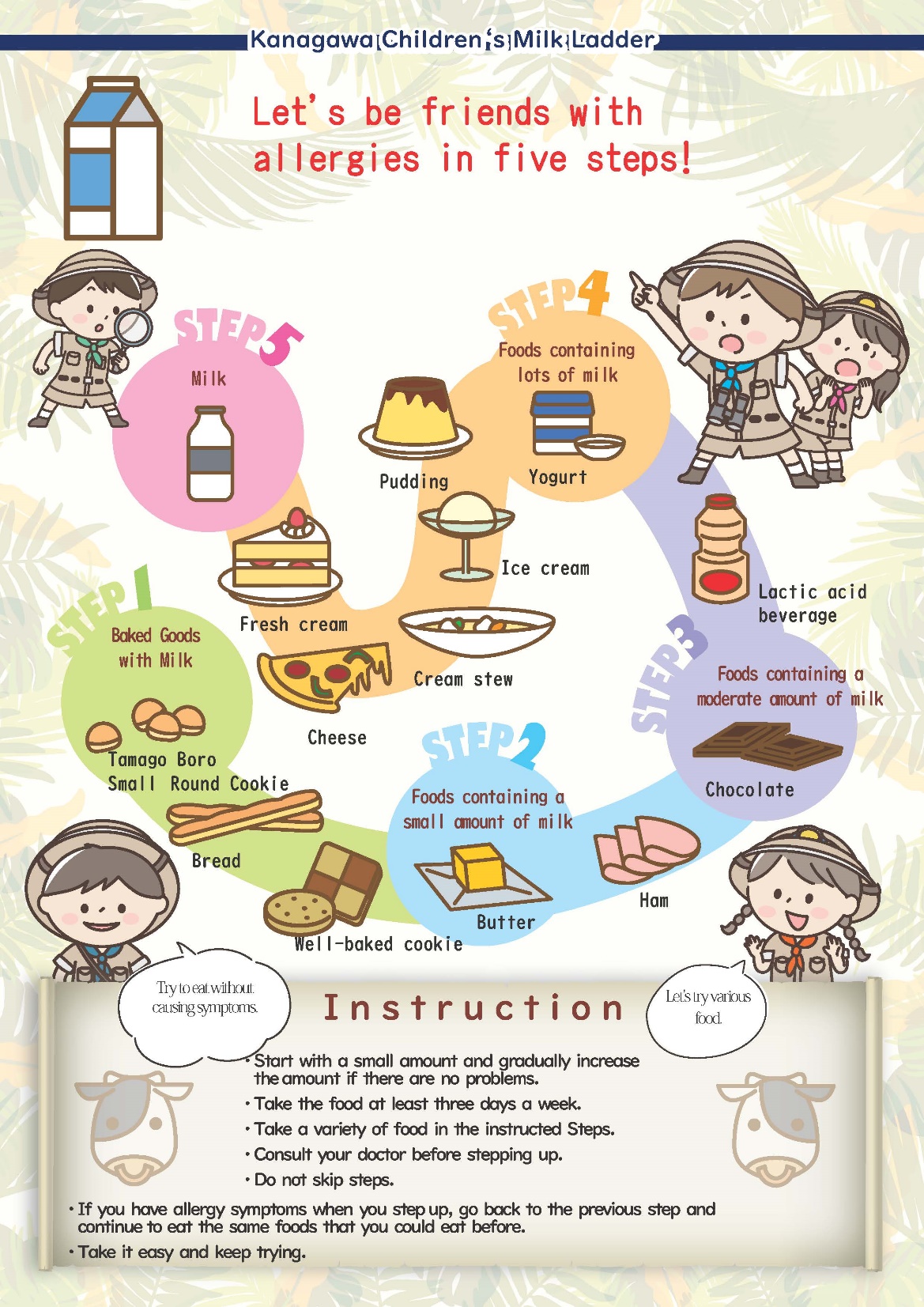


**Supplementary Figure** 1. Tell them to start with a small amount and gradually increase it if there are no problems. Consume a variety of foods at the steps indicated. If allergy symptoms occur, return to the previous amounts and steps and continue eating the same foods as before without pressure.


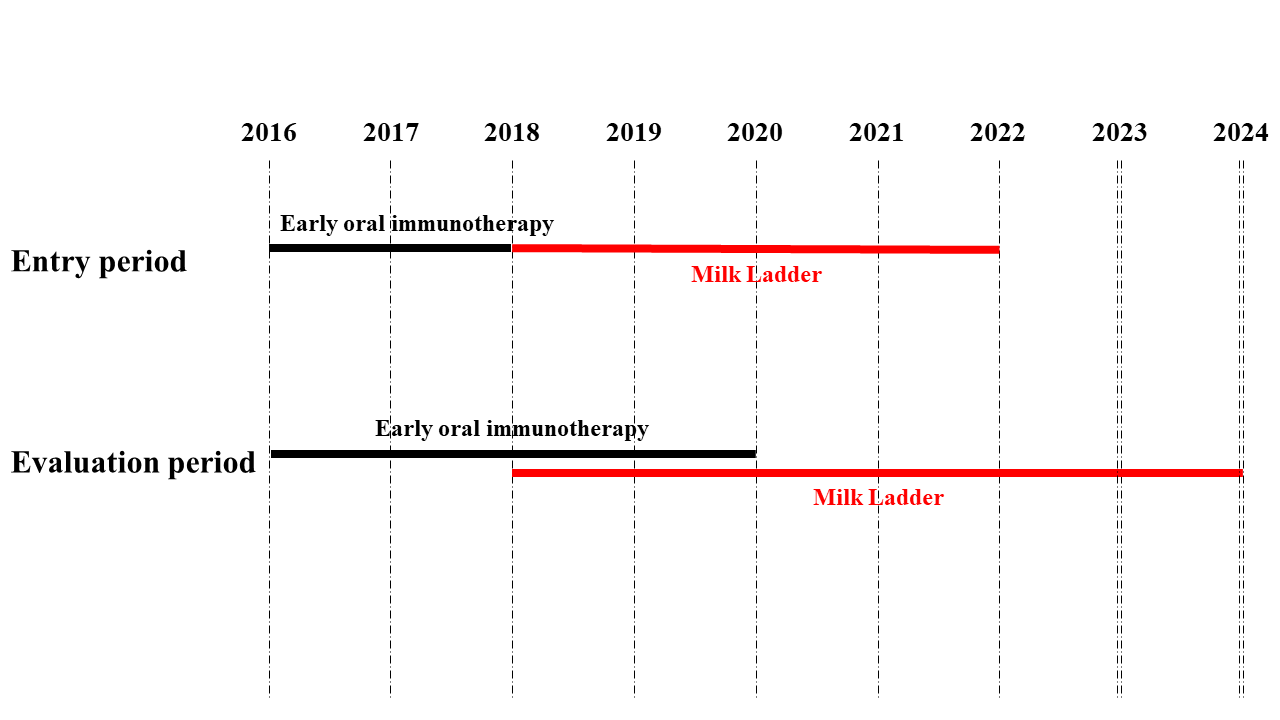


**Supplementary Figure 2**. The figure is a timeline showing the duration of patient inclusion and the duration of each patient's 2-year follow-up.
